# Climate change effects on airborne pathogenic bioaerosol concentrations: a scenario analysis

**DOI:** 10.1007/s10453-016-9435-5

**Published:** 2016-03-24

**Authors:** J. P. G. van Leuken, A. N. Swart, P. Droogers, A. van Pul, D. Heederik, A. H. Havelaar

**Affiliations:** 1 Centre for Infectious Disease Control (CIb), National Institute for Public Health and the Environment (RIVM), P.O. Box 1, 3720 BA Bilthoven, The Netherlands; 2 Institute for Risk Assessment Sciences (IRAS), Faculty of Veterinary Medicine, Utrecht University, Utrecht, The Netherlands; 3 Future Water, Wageningen, The Netherlands; 4 Environment and Safety (M&V), National Institute for Public Health and the Environment (RIVM), Bilthoven, The Netherlands; 5 Emerging Pathogens Institute, University of Florida, Gainesville, FL USA

**Keywords:** *Coxiella burnetii*, Q fever, Temperature, Global radiation, Wind, Precipitation, Atmospheric dispersion modelling

## Abstract

The most recent IPCC report presented further scientific evidence for global climate change in the twenty-first century. Important secondary effects of climate change include those on water resource availability, agricultural yields, urban healthy living, biodiversity, ecosystems, food security, and public health. The aim of this explorative study was to determine the range of expected airborne pathogen concentrations during a single outbreak or release in a future climate compared to a historical climatic period (1981–2010). We used five climate scenarios for the periods 2016–2045 and 2036–2065 defined by the Royal Netherlands Meteorological Institute and two conversion tools to create hourly future meteorological data sets. We modelled season-averaged airborne pathogen concentrations by means of an atmospheric dispersion model and compared these data to historical (1981–2010) modelled concentrations. Our results showed that modelled concentrations were modified several percentage points *on average* as a result of climate change. On average, concentrations were reduced in four out of five scenarios. Wind speed and global radiation were of critical importance, which determine horizontal and vertical dilution. Modelled concentrations decreased on average, but large positive and negative hourly averaged effects were calculated (from −67 to +639 %). This explorative study shows that further research should include pathogen inactivation and more detailed probability functions on precipitation, snow, and large-scale circulation.

## Introduction

The 2014 Fifth Assessment Report of the U.N. Intergovernmental Panel on Climate Change (IPCC) has further increased scientific evidence for global climate change in the twenty-first century (IPCC 2014). It describes the anthropogenic influence on the earth’s climate, the observed warming of the atmosphere and oceans during the last century, and the warming still to be expected for the current century. The exact effects on temperature, precipitation patterns, and large-scale circulation patterns are, however, highly dependent on the amount of greenhouse gases emitted in the nearby future.

Climate change affects, both positive and negative, water resource availability, agricultural yields, urban healthy living, biodiversity, ecosystems, food security, and public health (Boxall et al. 2009; Godfray et al. 2010; Hitz and Smith 2004; Szwed et al. 2010). The latter includes the extent of spread of vectorborne, waterborne, and airborne infectious diseases (Semenza and Menne 2009).

In the current study, we focused on the effects of climate change on airborne transmission of pathogenic bioaerosols (such as airborne bacteria and viruses; Després et al. 2012), which are able to cause infections in humans and animals by penetrating into the alveoli (Stuart and Wilkening 2005; Wéry 2014). Modelling airborne pathogenic transmission was performed in several studies using atmospheric dispersion models (ADMs) (Van Leuken et al. 2015a). ADMs are mechanistic models, initially developed for pollutant dispersion modelling (Holmes and Morawska 2006), and describe the spatial and temporal particle spread as a function of meteorological conditions, such as wind speed, wind direction, temperature, atmospheric stability, global radiation, and humidity. The spatial range of pathogen transmission in the outdoor environment extends from less than a metre up to multiple kilometres and even more and is highly dependent on meteorological conditions that influence particle/pathogen dilution and pathogen inactivation (Jones and Harrison 2004).

The current paper is part of a series of studies on Q fever (Van Leuken et al. 2013; Van Leuken et al. 2015a, b; Ladbury et al. 2015), a disease caused by the bacterium *Coxiella burnetii*. From 2007 to 2010, the Netherlands have experienced the largest Q fever epidemic ever described with over 4000 human cases (Dijkstra et al. 2012). Infected goats and sheep were associated with the epidemic with the airborne pathway being the main route of transmission. The epidemic caused approximately 497 disability-adjusted life years (DALYs) per 1000 symptomatic cases, while in comparison the 2009 influenza epidemic caused eight times less DALYs per 1000 symptomatic cases (Brooke et al. 2014).

The aim of this study was to investigate the range of expected airborne pathogen concentrations during an outbreak or release in a future climate compared to a historical climatic period (1981–2010). To that end, we used a climate change scenario for the period 2016–2045 (“Scenario-2030”) and four scenarios for the period 2036–2065 (“Scenario-2050”) as defined by the Royal Netherlands Meteorological Institute (KNMI) (Van den Hurk et al. 2014). They regard changes in temperature, precipitation, wind speed, and global radiation.

The results of this explorative study may give insight into the possible expected change in airborne pathogen concentrations during outbreaks or releases in a future climate and possible effects on infection pressure of airborne pathogenic bacteria and viruses.

## Method

### Concentration modelling

Van Leuken et al. (2015b) described the application of an atmospheric dispersion model to the Dutch Q fever outbreak, namely the OPS-ST (Operational Priority Substances—Short Term) model (version 10.3.2). This model was developed by the Netherlands National Institute for Public Health and the Environment (RIVM) to describe the transport of gases and particles in the atmosphere (Van der Swaluw et al. 2011; Van Jaarsveld and Klimov 2011; Van Jaarsveld 2004). The OPS-ST model includes wet and dry deposition, but no (pathogen) inactivation. We assumed a steady-state emission strength and used all other configurations from Van Leuken et al. (2015b), i.e. the modelled concentrations were not calibrated using measured *C. burnetii* concentrations (since those were not available); rather, the model was validated with Q fever case notifications. Pathogens were represented by particulate matter (PM_10_).

To determine the actual concentration change due to climate change effects, we investigated the change in concentration at:

1. A static receptor point, where we read the modelled concentrations. This receptor point is not directly linked to exposure of individuals, but rather a convenient proxy for population exposure, resulting from emissions from a single source. Thus, we considered 30-year climatic periods to investigate the total variation in time. We assumed a steady-state wind direction (270°) and put the receptor point 1 km downwind.
2. A spatial area of size 20 × 20 km with grid cells of 500 m in size. Since the aim of this analysis was to investigate spatial heterogeneity where we focused on seasonal-averaged concentrations in a single year, namely the Q fever year 2009 and reference years 2044 and 2064.

We downloaded historical meteorological data from the KNMI server for the Dutch meteorological reference station De Bilt (KNMI 2015). We then compared modelled concentrations per climate scenario (next section) to modelled concentrations of a historical climatic period (1981–2010) as a function of season and as a function of changes in temperature, wind speed, precipitation, and global radiation. We did not assume any other future developments, such as urbanisation.

### Climate scenarios

The Royal Netherlands Meteorological Institute (KNMI) defined climate scenarios for the periods 2016–2045 (Scenario-2030), 2036–2065 (Scenario-2050), and 2071–2100 (Scenario-2085) (Van den Hurk et al. 2014). We focused on Scenario-2030 and four sub-scenarios of Scenario-2050. The sub-scenarios are based on the amount of greenhouse gas emitted during the next decades and the possible effects of climate change on large-scale atmospheric pressure/circulation patterns:

- Scenario-2050-I: moderate temperature rise and limited changes in large-scale circulation patterns;
- Scenario-2050-II: moderate temperature rise and large changes in large-scale circulation patterns;
- Scenario-2050-III: high temperature rise and limited changes in large-scale circulation patterns;
- Scenario-2050-IV: high temperature rise and large changes in large-scale circulation patterns.

Table 1 summarises the projected changes of temperature, precipitation, wind speed and global radiation (based on Tables 4.1 and 4.2 in Van den Hurk et al. (2014)). Temperature and precipitation changes were defined for all seasons. Changes in wind speed (winter only) and global radiation (summer only) were defined for a single season. Changes in local wind directions were not taken into consideration following (Van den Hurk et al. 2014). We used the meteorological definition for seasons, i.e., winter, spring, summer, and autumn start on the 1st of December, March, June, and September, respectively.

**Table 1:** KNMI climate scenarios for the Netherlands for 2030 and 2050 [from Tables 4.1 and 4.2 in (Van den Hurk et al. 2014)]

Season	Variable	Climate 1981–2010 (reference)	Natural variation (90 %)	Scenario 2030	Scenario 2050-I	Scenario 2050-II	Scenario 2050-III	Scenario 2050-IV
Winter	Temperature	3.4 °C	±0.48 °C	+1.2 °C	+1.1 °C	+1.6 °C	+2.1 °C	+2.7 °C
	Precipitation	211 mm	±8.3 %	+8.5 %	+3.0 %	+8.0 %	+8.0 %	+17.0 %
	Wet hours (≥ 0.1 mm)	55 d	±4.7 %	+1.5 %	−0.3 %	+1.4 %	−0.4 %	+2.4 %
	Wind speed	6.9 m/s	±3.6 %	+0.5 %	−1.1 %	+0.5 %	−2.5 %	+0.9 %
Spring	Temperature	9.5 °C	±0.24 °C	+0.8 °C	+0.9 °C	+1.1 °C	+1.8 °C	+2.1 °C
	Precipitation	173 mm	±8.0 %	+5.5 %	+4.5 %	+2.3 %	+11.0 %	+9.0 %
Summer	Temperature	17.0 °C	±0.25 °C	+0.9 °C	+1.0 °C	+1.4 °C	+1.7 °C	+2.3 °C
	Precipitation	224 mm	±9.2 %	+0.2 %	+1.2 %	−8.0 %	+1.4 %	−13.0 %
	Max. hourly precipitation intensity	15.1 mm/h	±14 %	+8.25 %	+8.25 %	+10.5 %	+17.5 %	+19.0 %
	Wet hours (≥ 0.1 mm)	43 d	±6.4 %	+0.5 %	+0.5 %	−5.5 %	+0.7 %	−10.0 %
	Global radiation	153 kJ/cm^2^	±2.4 %	+1.9 %	+2.1 %	+5.0 %	+1.0 %	+6.5 %
Autumn	Temperature	10.6 °C	±0.27 °C	+1.0 °C	+1.1 °C	+1.3 °C	+2.2 °C	+2.3 °C
	Precipitation	245 mm	±9.0 %	+5.5 %	+7.0 %	+8.0 %	+3.0 %	+7.5 %

### Time-series conversion

The historical climatic period 1981–2010 served as the reference climatic period (Van den Hurk et al. 2014). Thus, we used these reference meteorological data in combination with the projected changes (Table 1) to create future meteorological data sets. The data in Table 1 are, however, expected *means*, whereas in reality these mean changes will be the result of a series of “extreme” conditions. Therefore, we used two time-series conversion methods developed by KNMI to create future 30-year hourly meteorological time series based on the period 1981–2010 and adjusted for the expected mean change and a variable’s natural variation (Bakker and Bessembinder 2012).

We applied the first method, originally developed for temperature conversion, to temperature, global radiation and wind speed. Firstly, the percentiles (10th, 50th and 90th) of variable *V* in a decade *δ* of 10 days in the future climatic period were based on the percentiles of the variable in the decade of 10 days in the historical climatic period and the percentile of the expected change of *V* due to climate change (Δ*V*):

1 $$\varPi_{{V^{\prime } ,\delta }}^{p} = \varPi_{V,\delta }^{p} + \varPi_{{\Delta V}}^{p}$$

where *Π*_*V*,*δ*_^*p*^ is the *p*th percentile of hourly data of variable *V* for the decade *δ.* Here, a decade is a meteorological expression and is defined as a sequence of 8, 9, 10, or 11 days (a month is subdivided into three decades with decade 1 including days 1–10, decade 2 days 11–20, and decade 3 the rest).

Variable *V*′ represents values of *V* in the future climatic period. Note that for all variables changing with a rate (%), the plus sign in Eq. (1) is replaced by a multiplication sign (Table 1).

Subsequently, each future hourly value (*V*′_*t*_) is derived from the historical hourly data (*V*_*t*_) with *t* being the index value of time in both climatic periods:

2 $$V_{t}^{\prime } = \varPi_{{V^{\prime } }}^{50} + \alpha \left( {V_{t} - \varPi_{V}^{50} } \right)$$

where *α* is a scaling factor equal to:

3 $$\alpha = \left\{ {\begin{array}{*{20}c} {\frac{{\prod_{{V^{\prime } }}^{90} - \prod_{{V^{\prime } }}^{50} }}{{\prod_{V}^{90} - \prod_{V}^{50} }}} &\quad {V_{t} \ge \prod_{V}^{50} } \\ {\frac{{\prod_{{V^{\prime } }}^{10} - \prod_{{V^{\prime } }}^{50} }}{{\prod_{V}^{10} - \prod_{V}^{50} }}} & \quad{V_{t} < \prod_{V}^{50} } \\ \end{array} } \right.$$

Transformation of historical precipitation data to future climatic periods was based on the second KNMI transformation method (Bakker and Bessembinder 2012). Firstly, the yearly summer maximum hourly precipitation intensities were adjusted according to the mean change (Table 1). Subsequently, we changed the number of “wet hours” in winter and summer by adding or removing precipitation events before and after historical precipitation sequences. The precipitation intensity and duration of new wet hours were set equal to the historical seasonal precipitation rate and duration of all wet hours per season. Thirdly, the precipitation intensity of all wet hours was changed according to the mean change in precipitation amount (corrected for the changes under the two prior steps).

## Results

**Fig. 1:**
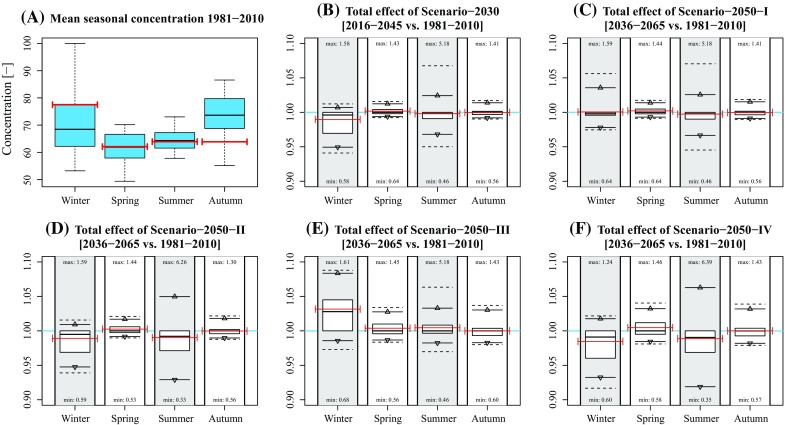
*Boxplots* of concentrations in 1981–2010 and the projected changes in 2016–2045 and 2036–2065. **a** *Boxplots* of seasonal daily averaged concentrations based on modelled hourly concentrations in the historical climatic period 1981–2010. **b**–**f** Change in hourly averaged concentrations as a result of climate change (Scenarios 2030, 2050-I, 2050-II, 2050-III, and 2050-IV). The *box* represents the interquartile distance (Q1–Q3) with the *bold line* representing the mean. The *lines* accompanied with the *triangle* represent the 2.5 % and 97.5 % quantiles. The *dashed lines* represent the 1 % and 99 % quantiles. Numbers near the *boxplots* represent the minimum and maximum rates per season. The *red lines* represent the years 2009, 2044, and 2064

Figure 1 shows boxplots of the modelled mean seasonal concentration of the historical climatic period 1981–2010 (subplot A), and the variability of the seasonal modelled change in concentration given the five scenarios at a single receptor point. None of the scenarios results in a significant mean change in concentration, and only some small changes can be observed in spring, summer, and autumn under Scenario-2030 and Scenario-2050-I.

**Fig. 2:**
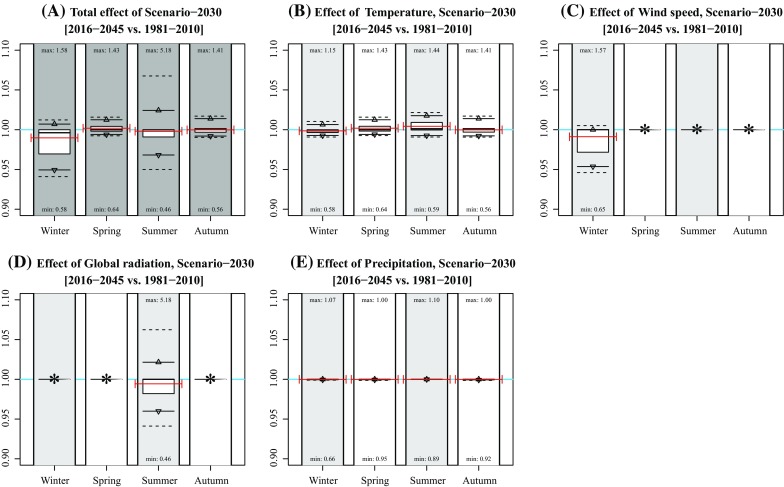
Effect of individual variables on projected concentration in Scenario-2030. **a** Idem as Fig. 1b; **b**–**f** idem as subplot A, but based on modified concentrations of changing single variable data (temperature, wind speed, global radiation, and precipitation). Numbers near the *boxplots* represent the minimum and maximum rates per season. The *red lines* represent the mean concentration (**a)** and mean rates of changes (**b**–**f)** of the years 2009, 2044, and 2064. *Asterisks* indicate that the effect was not included for that season

**Fig. 3:**
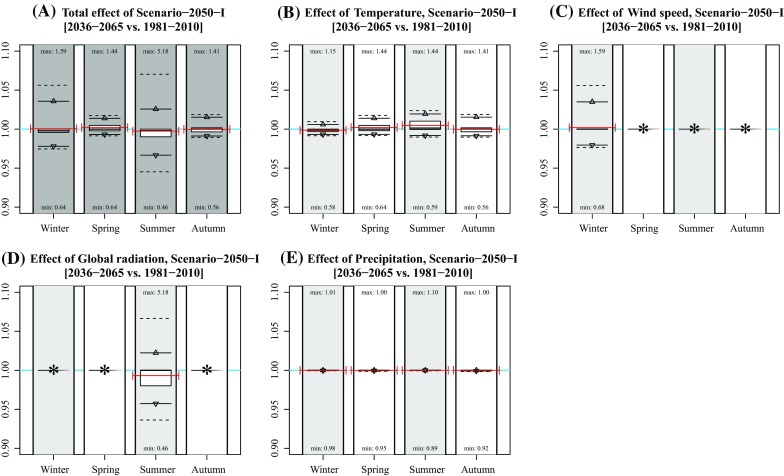
Effect of individual variables on projected concentration in Scenario-2050-I. **a** Idem as Fig. 1c; **b**–**f** idem as subplot A, but based on modified concentrations of changing single variable data (temperature, wind speed, global radiation, and precipitation). Numbers near the *boxplots* represent the minimum and maximum rates per season. The *red lines* represent the mean concentration (**a**) and mean rates of changes (**b**–**f**) of the years 2009, 2044, and 2064. *Asterisks* indicate that the effect was not included for that season

**Fig. 4:**
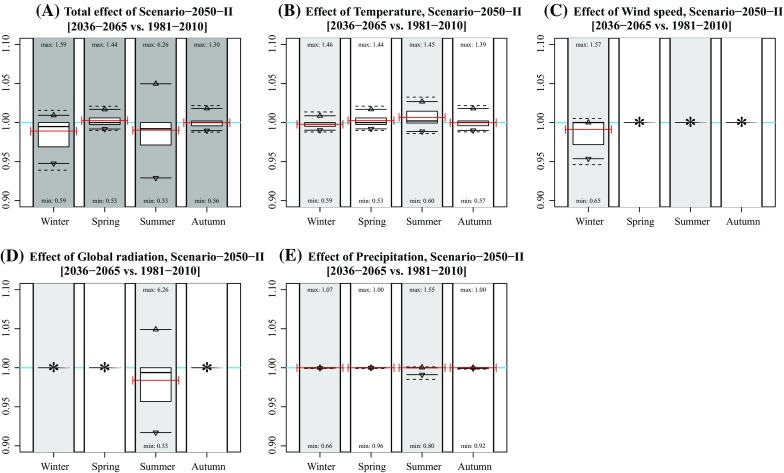
Effect of individual variables on projected concentration in Scenario-2050-II. **a** Idem as Fig. 1d; **b**–**f** idem as subplot A, but based on modified concentrations of changing single variable data (temperature, wind speed, global radiation, and precipitation). Numbers near the *boxplots* represent the minimum and maximum rates per season. The *red lines* represent the mean concentration (**a**) and mean rates of changes (**b**–**f**) of the years 2009, 2044, and 2064. *Asterisks* indicate that the effect was not included for that season

**Fig. 5:**
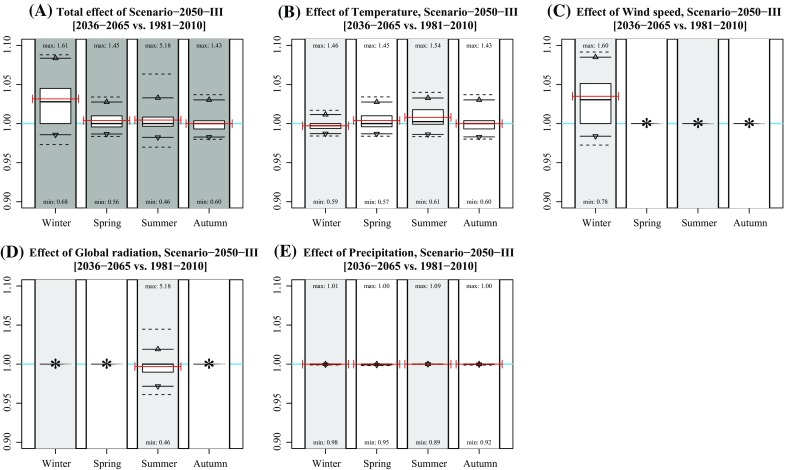
Effect of individual variables on projected concentration in Scenario-2050-III. **a** Idem as Fig. 1e; **b**–**f** idem as subplot A, but based on modified concentrations of changing single variable data (temperature, wind speed, global radiation, and precipitation). Numbers near the *boxplots* represent the minimum and maximum rates per season. The *red lines* represent the mean concentration (**a**) and mean rates of changes (**b**–**f**) of the years 2009, 2044, and 2064. *Asterisks* indicate that the effect was not included for that season

**Fig. 6:**
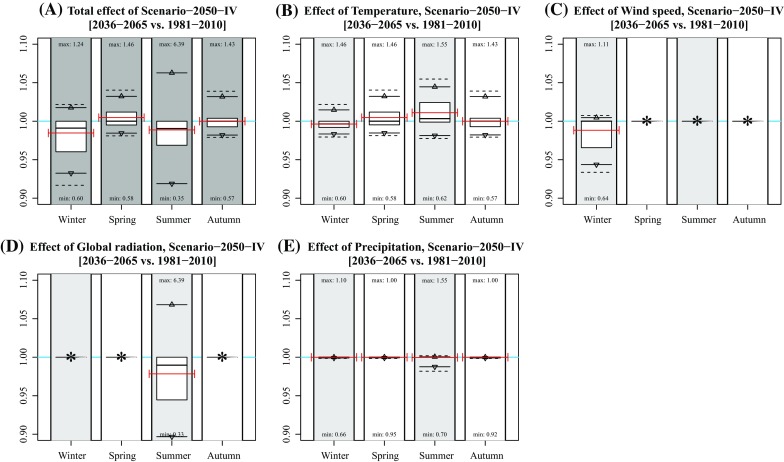
Effect of individual variables on projected concentration in Scenario-2050-IV. **a** Idem as Fig. 1f; **b**–**f** idem as subplot A, but based on modified concentrations of changing single variable data (temperature, wind speed, global radiation, and precipitation). Numbers near the *boxplots* represent the minimum and maximum rates per season. The *red lines* represent the mean concentration (**a**) and mean rates of changes (**b**–**f**) of the years 2009, 2044, and 2064. *Asterisks* indicate that the effect was not included for that season

Considering the 95 % intervals, the effects in winter and summer are generally largest. In winter, the largest effects occur under Scenarios 2030 [−5.1; 0.7 %], 2050-II [−5.2; 0.9 %], 2050-III [−1.4; 8.4 %], and 2050-IV [−6.8; 1.8 %], which is mainly caused by changing wind speeds (Figs. 2, 3, 4, 5, 6). The largest effects in summer occur under Scenarios 2050-II [−7.1; 5.0 %] and 2050-IV [−8.1; 6.3 %], mainly caused by changes in global radiation. The mean effect of temperature and precipitation is small.

In general, mean changes are limited up to several percentage points. Note that the effects are both positive and negative due to each variable’s natural variation (Table 1). The effect of climate change at individual hours is, however, much larger, given the maximum and minimum hourly changes in Fig. 1. Largest effects occur in summer with a change in concentration up to 639 % (Scenario 2050-IV, summer).

Table 2 shows the mean, 95 % interval and maximum and minimum change in seasonal-averaged concentration in an area of 20 × 20 km. In fact, the spatial change in concentration is rather heterogeneous: although the mean concentration does not change significantly, concentrations in individual grid cells decrease up to 20 % and increase up to 31 % at some places.

**Table 2:** Mean seasonal spatial change in concentrations with respect to reference year 2009 per scenario, based on a spatial area of 20 × 20 km

Scenario	Season	Mean (%)	Minimum (%)	2.5 percentile (%)	97.5 percentile (%)	Maximum (%)
2030	Winter	−0.5	−14.5	−7.2	0.1	1.7
2030	Spring	0.5	−9.5	−6.9	7.8	13.3
2030	Summer	−0.2	−14.9	−8.0	10.0	13.4
2030	Autumn	−0.6	−3.1	−2.3	10.6	14.1
2050-I	Winter	0.2	−15.1	−6.7	3.9	10.7
2050-I	Spring	0.8	−3.7	−2.7	9.0	12.6
2050-I	Summer	−0.2	−14.8	−8.0	10.0	13.5
2050-I	Autumn	−0.6	−2.5	−2.1	10.5	14.0
2050-II	Winter	−0.6	−14.5	−7.1	0.0	1.7
2050-II	Spring	0.3	−7.7	−6.1	9.3	13.3
2050-II	Summer	0.2	−14.2	−10.2	11.9	14.3
2050-II	Autumn	0.0	−6.2	−4.5	3.7	6.2
2050-III	Winter	5.6	−19.4	−12.0	13.8	31.3
2050-III	Spring	0.7	−3.1	−2.0	8.8	12.4
2050-III	Summer	−0.5	−18.7	−11.0	7.7	10.2
2050-III	Autumn	0.4	−9.0	−6.0	7.0	10.5
2050-IV	Winter	−1.1	−14.4	−7.3	−0.4	0.4
2050-IV	Spring	0.4	−3.9	−3.2	8.8	12.4
2050-IV	Summer	0.2	−14.9	−10.4	11.4	16.6
2050-IV	Autumn	−0.7	−11.0	−7.9	7.7	10.1

## Discussion

### Background

The effect of climate change on pathogen behaviour was investigated in several studies (e.g., Dukes et al. 2009; Sterk et al. 2013; Danovaro et al. 2009; Koelle et al. 2005; Hellberg and Chu 2015). However, these studies mainly focused on foodborne and waterborne pathogens. To our knowledge, the effect of climate change on concentrations of airborne pathogen has not been investigated yet.

That might, however, be relevant as outbreaks and releases of pathogens via the airborne transmission route do occur from a variety of sources including livestock, wastewater treatment plants, humans, and industry, either continuously or intermittently (Van Leuken et al. 2015a). In this study, we investigated the effects of climate change on airborne pathogen concentrations modelled using an atmospheric dispersion model.

### Interpretation

The single receptor results showed that modelled concentrations were modified (on average decreased) several percentage points *on average* as a result of climate change. In general, the variables wind speed and global radiation were of most importance, by influencing atmospheric particle dilution. An increase in global radiation (and temperature) enhances vertical atmospheric mixing and thus results in lower surface concentrations. An increase in wind speed enhances horizontal spread, and thus, the concentration at a receptor points at the plume axis (as in our study) decreases. From our spatial analysis, we concluded that distribution of the area at risk, however, changed: in some areas, the seasonal-averaged concentrations decreased (up to 20 %), while in others the concentrations increased (up to 31 %).

Essentially, the temporal coincidence of emission and specific meteorological conditions is crucial for the degree of exposure. After all, the occurrence of a shower can make a difference between no, limited, or full exposure. However, this is not different from current conditions. Therefore, our results indicated the probability that certain exposure events will occur more frequently or less frequently. On average, the modelled concentrations do not change substantially, only in the order of a few percentage points. However, extreme changes were calculated during specific meteorological conditions. This is particularly interesting concerning infectious diseases.

The effect of modified concentrations on health outcome depends on the host-specific dose (as a function of variables including breathing volume, exposure time, particle sizes, and airway geometry (Rostami 2009)) and pathogen-specific and host-specific response effects (Teunis and Havelaar 2000). As mentioned in the Methods section, the current study is part of a series of studies investigating the spatial transmission of *C. burnetii* that caused large Q fever epidemics in the Netherlands in 2009. Therefore, we added red bars in Figs. 1–6 representing years 2009 (and 2044 and 2064) to determine to what extent modelled concentrations in 2009 were exceptional. They show that the 2009 concentrations were rather average in spring and summer, but relatively high in winter and relatively low in autumn. Given the fact that most human infections occurred in spring, the 2009 concentrations were not exceptional: had the outbreaks occurred in years during which meteorological conditions favoured higher concentrations, the number of humans infected might even have been higher.

### Recommendations for further research

In this explorative study, we investigated the possible effects of climate change on modelled concentrations of airborne pathogens, represented by particulate matter (PM_10_). We used existing tools to convert observed meteorological data from a historical climatic period to future hourly values given a mean expected change of four variables and their natural variation. There are, however, several factors that may be very crucial and that might be incorporated or be improved:

1. Precipitation predictions: we used a relatively simple precipitation conversion tool developed by KNMI (Bakker and Bessembinder 2012). Precipitation, however, largely influences the particle deposition rate (Van Jaarsveld 2004). The absence or occurrence of precipitation is of much more influence than the actual change in precipitation intensity by climate change. Development of precipitation probability curves might improve the concentration prediction, possibly by applying a Monte Carlo approach.
2. Pathogen inactivation, although of critical importance (Jones and Harrison 2004), was not considered.
3. Effect of snow cover was not considered either, although its effect on (modelled) concentrations is rather large. Firstly, our atmospheric dispersion model assumes a reduction of the surface roughness length to 5 cm in case of a snow cover (whereas 28 cm is the mean roughness length in our reference area) (Van Jaarsveld 2004). Secondly, a snow cover causes the air layer above to cool down, thereby causing it to become more stable. As a result, vertical atmospheric mixing is reduced or prevented, and surface concentrations remain high. Therefore, it would be recommendable using or creating a more detailed snow prediction model including the climate change parameters for precipitation and temperature.
4. Inclusion of circulation patterns: we assumed a constant wind direction to determine the change of concentration at a single receptor point. However, by including actual wind directions, geospatial plots could be created with expected differences in concentrations. Moreover, by including (probability curves of) expected changes in circulation patterns, the effect of large-scale circulation patterns may be quantified as well (although the actual location of a source will be more determinant).

## Conclusions

Effects of climate change are more likely to be observed when considering long-term meteorological data sets (Marotzke and Forster 2015). In this study, we used 30-year observed and projected meteorological data to quantify the effect of climate change to airborne pathogen concentration for the periods 2016–2045 and 2036–2065. We concluded that for four out of five scenarios the concentrations generally decrease as a result of increased global radiation, temperature and increased wind speeds, whereas for one scenario the concentrations generally increase. Nevertheless, the differences between and especially within seasons are large. Since coincidence of emission and specific meteorological conditions largely determines the actual exposure, additional investigations are required to further quantify the change in predicted concentrations of airborne pathogenic bioaerosols by taking into account pathogen inactivation and more detailed probability functions on precipitation, snow and large-scale circulation.
